# Naturalized Dyes: A New Opportunity for the Wood Coloring

**DOI:** 10.3390/polym15173632

**Published:** 2023-09-01

**Authors:** Laura Vespignani, Marco Bonanni, Marco Marradi, Benedetto Pizzo, Roberto Bianchini, Giacomo Goli

**Affiliations:** 1 Department of Chemistry “Ugo Schiff”, University of Florence, Via della Lastruccia 3-13, 50019 Sesto Fiorentino, Italy; marco.bonanni@unifi.it (M.B.); marco.marradi@unifi.it (M.M.);; 2 Department of Agriculture, Food, Environment and Forestry (DAGRI), University of Florence, Piazzale delle Cascine 18, 50144 Florence, Italy; giacomo.goli@unifi.it; 3 CNR-Institute of Bioeconomy (IBE CNR), Via Madonna del Piano, 10, 50019 Sesto Fiorentino, Italy; benedetto.pizzo@ibe.cnr.it

**Keywords:** naturalized dyes, wood dyeing, UV-Vis analysis, color fastness

## Abstract

Naturalized dyes (NDs) are innovative and eco-friendly synthetic compounds in which a chromophore is covalently linked to a natural sugar (e.g., lactose). The sugar moiety confers water-solubility and biocompatibility to the dye molecule as a whole. NDs have demonstrated potential application in dyeing textiles and leather. The purpose of this work was to demonstrate that selected NDs can be also applied to dye wood. To that aim, two NDs were tested to color beech and poplar wood. The NDs were applied as a simple aqueous solution or mixed with a waterborne, biogenic staining agent (commercially available Gemma U50). Moreover, the effect of the application of a biogenic waterborne top coat (commercially available Resina Plus U49) was also studied. Different methods were tested to investigate the potential application of these NDs to wood. The dyeing behavior was analyzed in terms of penetration into the substrate, covering capacity and color homogeneity through macro- and microscopic observations and colorimetric measurements. The color fastness to water washout and the color stability to light, in particular by exposing the wooden samples to artificial aging (UV radiations in a Solar Box), were also investigated. The NDs, when used as water solutions, were able to afford a homogeneous coating and a pleasant appearance on the wood surface, as well as a good color fastness to washout with water. Dissolving the dyes in the stain or applying the top coat generally resulted in even better color fastness to washout. However, all the application methods tested showed limited resistance to fading in the Solar Box, which therefore remains a drawback for this type of product.

## 1. Introduction

Dyeing is a widespread practice that was introduced by humans more than 30,000 years ago [1] in order to improve the aesthetics of textiles. Natural dyes were the sole source of color until 1856 when the first human-made organic aniline was synthesized [2].

Today, there are many types of synthetic dyes available on the market, and most of them are disperse dyes [3]. Disperse dyes are small organic molecules of non-ionic nature, scarcely soluble in water, such as anthraquinones or azo compounds. The dispersions of these dyes in the dyeing bath are usually achieved with the aid of chemical auxiliaries. Auxiliaries are mainly additives such as heavy metals, dyeing carriers, surfactants, dispersants and other organic molecules, which improve the stability and the performance of the dyeing suspensions, but end up, for the most part, in wastewater [4,5,6,7]. The heterogeneity and large number of auxiliary compounds used, along with their toxicity and poor biodegradability, make the treatment of dyeing wastewater very difficult and expensive [8,9,10,11] resulting in an unsustainable environmental footprint. Moreover, inefficient textile dyeing processes can cause the release of part of the dyes into the wastewater. It is known that azo dyes, which represent more than 60% of the disperse dyes on the market, have toxic effects on both the ecosystem and human health. Fish, humans and other living organisms can be exposed to azo dyes through ingestion or direct skin contact. Inside the body, these compounds are metabolized into toxic intermediates, which have a negative impact on several tissues and organs, and, in some cases, their degradation produces carcinogenic amines [12].

Natural dyes provide acceptably good color fastness to water washing when applied to various textiles; however, they are usually limited in coloring wood surfaces because they can have a low affinity for binding to wood [13]. In these cases, mordants are normally used, which are able to form complexes with the dyes, thus increasing their binding to the wood surface. However, mordants are usually based on metals (e.g., iron, aluminum) or they are tannins or oils [14,15,16]. All of them can change the natural color of the dye, and in addition, some metal-based mordants can be toxic, harmful or produce toxic waste [17,18]. Moreover, natural dyes are usually obtained by the laborious extraction processes of the coloring component from the raw material [19,20]. Therefore, it becomes very important to explore possible ways to overcome these disadvantages; i.e., to find products with improved color fastness to water washout when applied to wood surfaces, the absence of any mordant and dyes that are easy to obtain and manage.

In the last ten years, naturalized dyes (NDs) have emerged as a new class of water-soluble and eco-sustainable dyes, thanks to the possibility of being applied without the addition of any toxic or impactful chemical auxiliaries [21]. Naturalization is achieved by glycoconjugation, i.e., by forming a chemical bond between a synthetic dye (e.g., azo, anthraquinone, aniline type of chromophore) and a natural sugar, for example, lactose, which is able to impart remarkable solubility in water and biodegradability to the dye molecule [22]. For this reason, NDs are also known as ‘glycoconjugate azo dyes’ (GADs) [23,24]. The range of synthetic dyes that can be subjected to glycoconjugation has been expanded and has attracted much attention because of the possibility of increasing susceptibility to microbiological degradation [25]. NDs have demonstrated their potential application in dyeing materials of different nature, such as textiles, hair and leather [26,27]; however, no examples of the use of these NDs in the wood sector was found in the literature.

It is well known that wood is a renewable material with a unique touch and feel, excellent physical and mechanical performances [28] and an outstanding environmental profile. The different wood species cover a wide range of natural colors, but very often the material is dyed for different reasons, such as to decrease the inhomogeneity of the wooden surface, mimic the appearance of more expensive species or obtain colors that do not exist in the range of the native color spectrum. Full volume dying is also the base of high-technology products such as multilaminar and decorative wood veneers, which are both used to mimic existing species or to create new decorative design products [29], with a special eye to sustainability.

**Figure 1:**
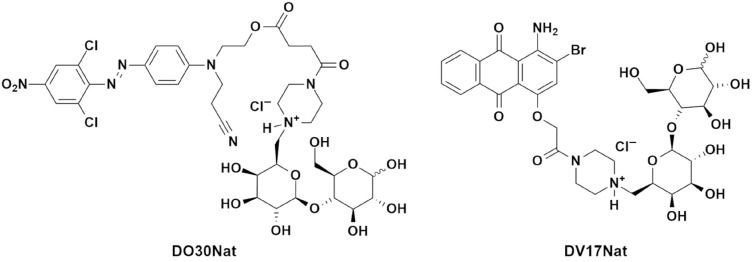
Chemical structures of the naturalized dyes (NDs) tested for the dyeing of beech and poplar wood: Naturalized Disperse Orange 30 (**DO30Nat**) and Naturalized Disperse Violet 17 (**DV17Nat**).

Designing new eco-friendly products for the field of woodworking has become of increasing interest in recent decades [30]. The possibility to develop new products based on renewable and responsibly sourced raw materials dyed with NDs has been investigated. Two synthetic and water-soluble NDs were tested in dyeing beech and poplar wood to explore the possible interest of this group of compounds for the wood industry; one naturalized anthraquinone-based dye **DV17Nat** (Naturalized Disperse Violet 17) and one naturalized azo dye **DO30Nat** (Naturalized Disperse Orange 30) [26,27]. The chemical structures of these dyes are reported in Figure 1.

In spite of the “azo” nature of its chromophore, **DO30Nat** was selected because it does not produce carcinogenic aromatic amines after degradation. In fact, Disperse Orange 30 is not part of the list of restricted substances reported in the REACH Regulation [31]. A previous study, where various possible bioremediation procedures for the removal of dyes from the wastewater of textile and leather dyeing industrial plants were investigated, confirmed the absence of dangerous aromatic amines in the degradation products of **DO30Nat** [32].

In order to carry out the wood dyeing, NDs were dissolved in water or in the waterborne and biogenic staining agent, based on drying oils emulsified with casein and soy lecithin (Gemma U50). These coloring solutions were applied to beech and poplar wood, both permeable and clear hardwoods, but with different porosity. Dip and superficial coloring with a dye-water solution were tested, as well as superficial coloring with the staining agent. Color fastness to water and UV resistance were also tested before and after the application of a top coat composed of drying oils emulsified with casein and soy lecithin (Resina Plus U49).

## 2. Materials and Methods

### 2.1. Dyes

Two naturalized dyes (NDs) were used for the tests: Disperse Orange 30 Naturalized (**DO30Nat**) and Disperse Violet 17 Naturalized (**DV17Nat**), which are depicted in Figure 1 and the synthetic routes of which are reported in the Supplementary Materials.

**DO30Nat** was synthesized from the crude press-cake chromophore Disperse Orange 30 (C.I.11119, CAS [12223-23-3], the chromophore of the commercially available Foron Brown Yellow S-2RFL 150, Cromatos SrL, Forlì, Italy), according to the naturalization process reported in [26] (Scheme S2). **DV17Nat** was synthesized after the isolation of chromophore Disperse Violet 17 (C.I. 60712, CAS [12217-92-4], the chromophore from commercially available Latyl Red B, Chimica Tessile S.r.l., Prato, Italy) through extraction with dichloromethane by using a Soxhlet apparatus [33], following the procedure described in the patent WO 2014/177528 A1 [26] (Scheme S3).

### 2.2. Wood Samples

Beech (*Fagus sylvatica* L.) and poplar (*Populus alba* L.) wood were chosen as benchmarks because of their light color and permeability. A total of 72 clear wood samples oriented according to the anatomical directions, with dimensions of 20 mm (R) × 20 mm (T) × 100 mm (L) per species, were prepared. The samples of beech and poplar wood were obtained from the same board in order to minimize wood variability and were planed on the four faces in a longitudinal direction. The density (ρ), determined after conditioning in a climatic chamber at 20 °C and 65% relative humidity (R.H.), of beech samples was (699 ± 47) kg/m^3^, while the ρ of poplar samples was (363 ± 65) kg/m^3^.

### 2.3. Wood Dyeing

#### 2.3.1. Dyeing with Aqueous Solution

Aqueous solutions of dye at a concentration of 2 g/L were prepared with deionized water (18.2 MOhm cm) using a Milli-Q system (Millipore, Bedford, MA, USA).

Three application methods in aqueous solution were studied: impregnation under vacuum (**Method 1**), dip coating with a single immersion (**Method 2**) and dip coating with two serial immersions (**Method 3**). For the three applications, the same bath was sequentially used after checking by UV-Vis analysis that no differences occurred in the bath concentration (Section 3.1).

***Method 1****:* The wood samples were immersed in the dyeing bath and a vacuum (−970 mbar) was made in the dyeing chamber in order to remove most of the air from the pores, after one-hour atmospheric pressure was restored and pressure cycle at 7.5 bar was applied for one hour in order to help the penetration of the color into the substrate. This treatment is designed for wood mass coloring or impregnating. Finally, the samples were removed from the dyeing baths, dabbed with a paper towel and conditioned in a controlled atmosphere cell at 20 °C and 65% R.H. until stabilization.

***Method 2****:* The wood samples were immersed in the dyeing baths for 20 s, pulled out for 5 s and immersed again for 20 s. Then, the samples were removed from the dyeing solution, dabbed with a paper towel and conditioned in a cell at 20 °C and 65% R.H. until stabilization.

***Method 3****:* This treatment was based on repeating the steps described for **Method 2** twice but placing the wood samples in the oven at 103 °C for 2 min between the two immersions in order to partially dry the wood and facilitate the color uptake. As described above, after dyeing, the samples were conditioned under a controlled environment at 20 °C and 65% R.H. until stabilization.

Each method was applied in quadruplicate: 4 beech samples and 4 poplar samples were used for each ND.

#### 2.3.2. Staining

The biogenic waterborne resin Gemma U50 (Solas s.a.s., Cernusco Lombardone, Italy) was chosen as the staining agent in order not to compromise the sustainability of the NDs. Indeed, Gemma U50 is a high-brightness water-based resin entirely made from renewable compounds, such as natural drying oils (e.g., linseed oil, stand oil, poppyseed oil), casein and soy lecithin. NDs were dispersed in Gemma U50, and the effect of different concentrations was tested (2 g/L, 4 g/L, 6 g/L, 8 g/L and 10 g/L). The dispersions were left at room temperature in a sealed flask for 1 month in order to verify the long-term stability (Section 3.2). The application method was performed following the Gemma U50 manufacturer’s recommendations.

***Method 4****:* The stains were prepared by dispersing the ND (**DO30Nat** and **DV17Nat**) in Gemma U50 (concentration of 2 g/L). Two layers of stain were applied by brush on 4 beech samples and 4 poplar samples for each ND. For each layer, the stain was applied in parallel non-overlapping strokes by using the same flat brush for all the samples. Each brushstroke was carried out in one go at constant speed, in order to have a coating as homogeneous and thin as possible.

#### 2.3.3. Application of the Clear Top Coat

Resina Plus U49 (Solas s.a.s., Cernusco Lombardone, Italy) is a water-based, uncolored top coat, composed of a mixture of natural vegetable oils and resins. It was used in the following way in agreement with the manufacturer’s requirements:

***Method 5:*** Two layers of Resina Plus U49 were applied by brush to 8 beech samples and 8 poplar samples, previously colored with **DO30Nat** and **DV17Nat** in water solution (2 g/L). For each layer, the coat was applied following the same procedure used for the stain in **Method 4** (Section 2.3.2).

### 2.4. Water-Based Dyeing Bath Stability over Time

During the application tests in aqueous solutions (Section 2.3.1), a few mL were taken from each dyeing bath at three different times: freshly prepared (***t*_0_**); after the dyeing tests with **Method 1** (***t*_1_**); after the dyeing tests with **Method 2** (***t*_2_**). Each sampling was diluted by 1 to 10 and analyzed by using a Varian Cary-Win 4000 UV-Vis spectrophotometer, measuring the absorbance in a range from 300 nm to 800 nm, to observe any variations in the concentration of the chromophore during the various impregnations. The absorbance spectra of ***t_0_***, ***t_1_*** and ***t_2_*** of each dyeing bath were compared (Section 3.1).

### 2.5. Dyeing Capacity

The final color of the wooden samples was assessed by colorimetry. A portable X-Rite SP60 spectrophotometer in specular component excluded mode was employed. For each sample, three measurements were carried out before and after the coloring treatment on a specific area previously located by using a mask. Results were elaborated and reported in the CIE L*a*b* standard color system, the most widely used color system within the wood industry today [34]. The L*a*b* system describes color as a rotational space where each color point is quantitatively represented by three coordinates. These three coordinates are: L* associated with the luminance, a* associated with the green-red axes and b* associated with the blue-yellow axes. In this system, different colors can be compared by the color distance, ΔE*, expressed as the Cartesian distance between two points in this color space. ΔE* is thus expressed as:

$$\Delta E^{*}=\sqrt{{\left(L_{1}^{*}-L_{2}^{*}\right)}^{2}+{\left(a_{1}^{*}-a_{2}^{*}\right)}^{2}+{\left(b_{1}^{*}-b_{2}^{*}\right)}^{2}}$$

ΔE* was calculated for each type of ND and application method; the subscripts 1 and 2 indicate the L*, a* or b* values after and before the application of the dye, respectively. The Metric Chroma (C*) and Metric Hue Angle (h) were also calculated for both untreated and dyed samples. C* is defined by the following formula:

$$C^{*}=\sqrt{{\left(a^{*}\right)}^{2}+{\left(b^{*}\right)}^{2}\;}$$

Hue angle formulas are different depending on which quadrant the color is located: the first quadrant [+a*,+b*], that goes from 0° (red) to 90° (yellow); the second quadrant [−a*,+b*], that goes from 90° (yellow) to 180° (green); the third quadrant [−a*,−b*], that goes from 180° (green) to 270° (blue); the fourth quadrant [+a*,−b*], that goes from 270° (blue) to 360° (red) [35]. Since all the a* and b* values obtained here were positive, the formula exploited was the following:

$$h\;\left({}^{\circ}\right)=\tan^{-1}\left(\frac{b^{*}}{a^{*}}\right)$$

Colorimetric measurements were also carried out on the poplar samples used for the solubility tests in Gemma U50 to quantify the covering capacity at different concentrations (Section 3.2).

ΔE* was calculated for each type of ND and application method.

The same method was carried out on the poplar samples used for the solubility tests in Gemma U50 to quantify the covering capacity at different concentrations (Section 3.2).

The samples treated by impregnation under vacuum (**Method 1**) and by dip coating (**Method 3**) were cross cut every 20 mm in length to obtain 5 small specimens to study the penetration of the dye into the core. To evaluate the ability of **Method 1** to color the wood in depth, on the cross face of the central specimen of each sample, three colorimetric measurements were taken and the color distance between them and the ones taken on the head of the sample before the coloring treatments was calculated. The ΔE* obtained for **Method 1** and **Method 3** were compared. Macro pictures were taken on the same face by using a Dino-Lite USB digital microscope.

### 2.6. Color Fastness to Water Washout

The final color resistance to water washout was studied by measuring the fastness grade (FG) of the colored surfaces. Since, currently, there are no standard methods for color fastness tests for wood, the UNI EN 646 [36] for cardboard was applied. In particular, the procedure for short-term contact used for dyed paper and board intended to come into contact with foodstuffs was followed. This method involved keeping two dyed surfaces per sample in contact with two glass fiber sheets, soaked in water, for 10 min and observing, after letting the sheets air dry, the eventual color migration over them. The determination of the color migration was made by the colorimetric difference between the initial and the final state of the same glass fiber sheet after being in contact with the sample. The measurement was made with an X-rite SP60 spectrophotometer using CIEL*a*b* coordinates. According to the standard used, a given ΔE* can be associated with an FG by using a conversion table [37]. The FG is defined by a scale of 9 values ranging from 1 to 5, where 1 symbolizes a very low color fastness and 5 is an optimal fastness. The measures were carried out on the samples treated with **Methods 3**, **4** and **5**, using **DO30Nat** and **DV17Nat** as chromophores. For each sample, five measurements per glass fiber sheet were carried out on the side kept in contact with the colored surface.

### 2.7. Color Fading in Aging Test

To observe the photosensitivity of the NDs, the samples treated with **Methods 3**, **4** and **5**, using **DO30Nat** and **DV17Nat** as chromophores, were subjected to artificial aging, together with control samples of both wood types. Then, 20 mm (R) × 20 mm (T) × 50 mm (L) specimens were obtained from the colored samples and half covered with aluminum foil to have, at the end of the test, a direct comparison between the original appearance of the wood and the one after exposure to artificial aging. The specimens remained for 50 h under a Xenon-arc lamp with a UV filter with a cut-off < 290 nm, in a CO.FO.MEGRA Solar Box3000e according to ISO 11341:2004 [38] (irradiance at 550 W/m^2^ and black standard temperature at 65 °C). On the face exposed to aging, colorimetric coordinates were recorded with an X-rite SP60 spectrophotometer before the test, after 6, 26 and 50 h of exposure. Each time, the CIEL*a*b* measurements were taken from three different points and then averaged. The color fading was quantified by calculating the ΔE* at 6, 26 and 50 h of exposure. As the colored coating is transparent and given the high photosensitivity of wood, ΔE** for the dyed samples after aging was calculated taking into account the changes in the color coordinates of the substrate using the following formula:

$$\Delta E^{**}=\sqrt{{\left(\Delta L_{c}^{*}-\Delta L_{r}^{*}\right)}^{2}+{\left(\Delta a_{c}^{*}-\Delta a_{r}^{*}\right)}^{2}+{\left(\Delta b_{c}^{*}-\Delta b_{r}^{*}\right)}^{2}}$$

ΔL*_c_, Δa*_c_ and Δb*_c_ indicate the difference between L*, a* or b* values registered on the dyed samples after and before the exposure. ΔL*_r_, Δa*_r_ and Δb*_r_ indicate the difference between L*, a* or b* values registered on the reference samples after and before the same time of aging.

## 3. Results and Discussion

### 3.1. UV-Vis Analysis on the Water-Based Dyeing Baths

**Figure 2:**
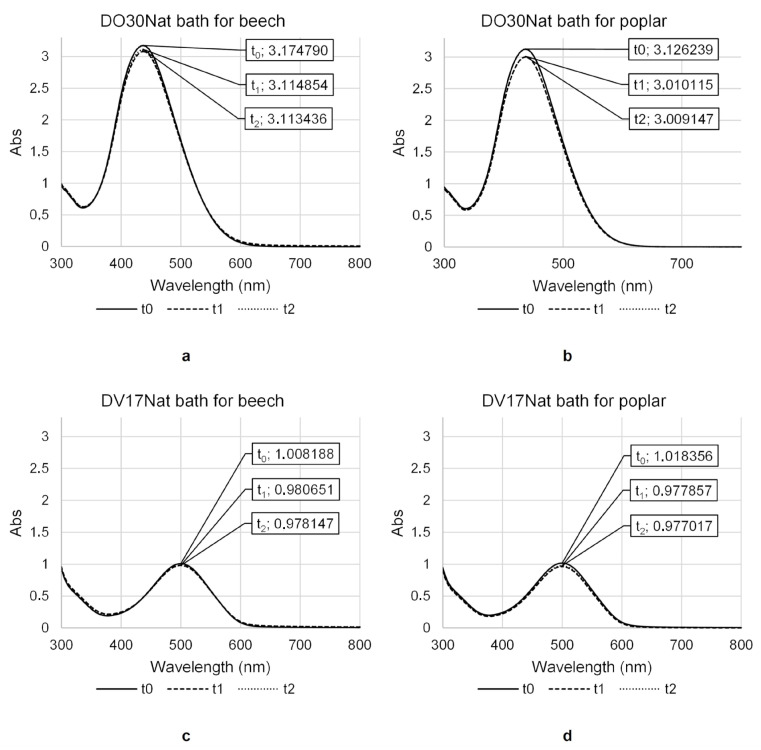
UV-Vis absorbance spectra recorded at ***t*_0_** (freshly prepared), ***t*_1_** (after the dyeing tests with **Method 1**, application of vacuum-pressure cycle in water coloring solutions) and ***t*_2_** (after the dyeing tests with **Method 2**, single immersion in water coloring solutions) specimens of **DO30Nat** (**a**,**b**) and **DV17Nat** (**c**,**d**) dyeing baths used to color beech (left) and poplar (right) samples.

**DO30Nat** and **DV17Nat** gave stable and optically isotropic solutions with water at a concentration of 2 g/L. Even after weeks, no color changes or phase separation were noticed in the dyeing baths. The UV-Vis spectra registered on the specimens taken from **DO30Nat** and **DV17Nat** baths freshly prepared (***t*_0_**), after the dyeing tests with **Method 1** (***t_1_***) and after the dyeing tests with **Method 2** (***t*_2_**), were compared (Figure 2).

A slight decrease in the absorbance values was observed between ***t*_0_** and ***t*_1_** specimens on the characteristic band of both **DO30Nat** (maximum absorption at 437 nm) and **DV17Nat** (maximum absorption at 500 nm). The absorbance variation seemed bigger in the baths used to dye the poplar samples, perhaps because poplar wood tends to absorb more liquid during the vacuum-pressure cycle treatment due to its lower density. However, the variations in concentration of the chromophore in solution are too small to be related to a reaction between the components of the wood and the NDs. Dyeing is more likely to occur by fixing the chromophore molecules into the porosity of the wood during water uptake. The difference in absorbance between ***t*_1_** and ***t*_2_** was not significant. This means that by immersing the wooden samples for a few minutes, the composition of the dyeing bath does not change and thus it is possible to reuse the same bath for several consecutive treatments before its exhaustion. This is a positive feature from an environmental point of view, as it implies a limited consumption of NDs to obtain a pleasant aesthetic appearance.

### 3.2. Effect of ND Concentration on Staining Agent

**DO30Nat** and **DV17Nat** gave a stable coloring dispersion with Gemma U50 resin (Section 2.3.2). Even at a concentration of 10 g/L of ND, no phase separation was observed after 1 month of storage. NDs have a similar behavior to that of surfactants, thus, it is likely that the presence of oily components in Gemma U50 aids the dispersion and improves its stability.

**Figure 3:**
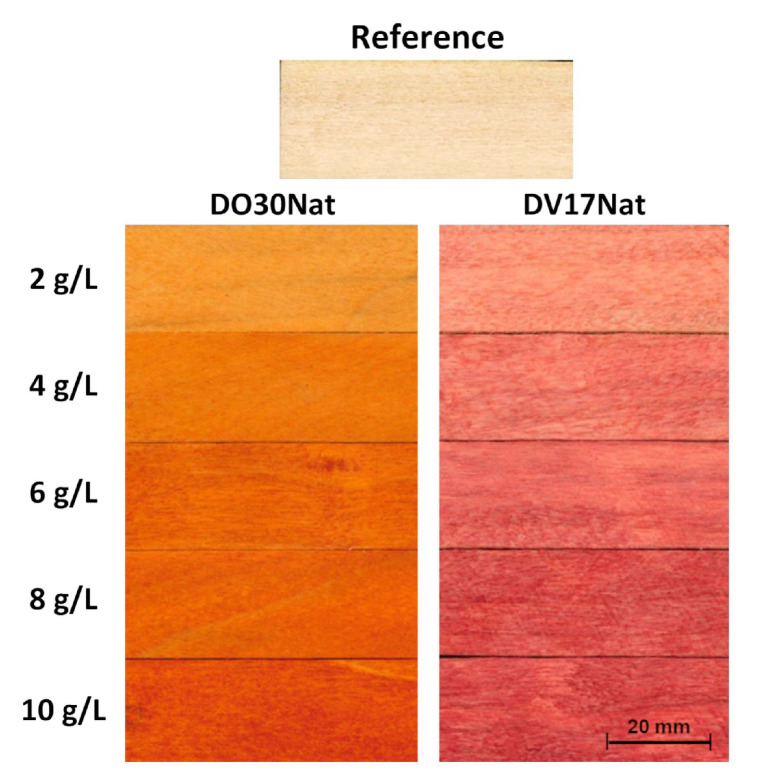
Macroscopical aspect of poplar samples dyed by applying one layer of Gemma U50 Solas^®^ mixed with **DO30Nat** (**left**) and **DV17Nat** (**right**) on the surface, by brush, at different concentrations (from the (**top**): 2, 4, 6, 8 and 10 g/L).

**Figure 4:**
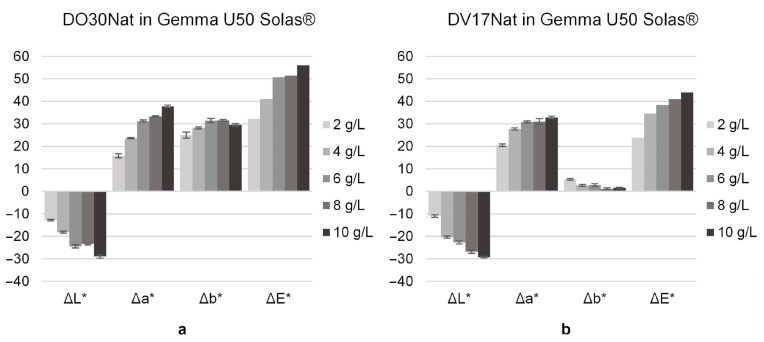
Color difference in terms of ΔL*, Δa*, Δb* and ΔE* between before and after the coloring treatment, measured on poplar samples dyed by applying on the surface, by brush, one layer of Gemma U50 Solas^®^ mixed with **DO30Nat** (**a**) and **DV17Nat** (**b**) at different concentrations (2, 4, 6, 8 and 10 g/L).

The color obtained by applying the stain with 2 g/L of ND showed a coverage and a vividness comparable to those obtained with the aqueous solutions. Moreover, Figure 3 shows the effect of using different ND concentrations on poplar wood. Above 4 g/L, the benefits in terms of color intensity are not readily apparent from the photographic comparisons. Colorimetric analysis confirmed that the further increase in concentration above 4 g/L resulted in smaller color variations (Figure 4).

### 3.3. Wood Coloring

**Figure 5:**
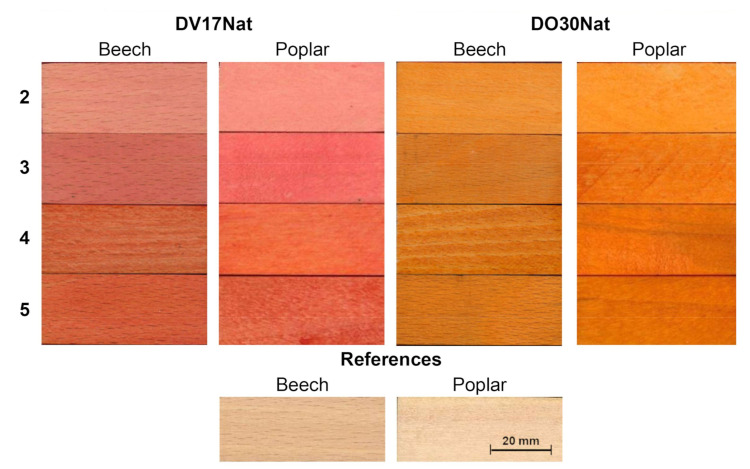
Macroscopical aspect of beech and poplar samples dyed with **DV17Nat** (**top left**) and **DO30Nat** (**top right**) following **Method 2** (single immersion in water coloring solutions), **Method 3** (serial immersion in water coloring solutions), **Method 4** (application of Gemma U50 Solas^®^ coloring dispersion by brush) and **Method 5** (application of Resina Plus U49 Solas^®^ on water-based colorations). Uncolored wooden references are also shown (**bottom**).

During the coloring tests with aqueous solutions, **DO30Nat** and **DV17Nat** gave, in general, vivid and covering colorations with all the application methods (Figure 5).

**Figure 6:**
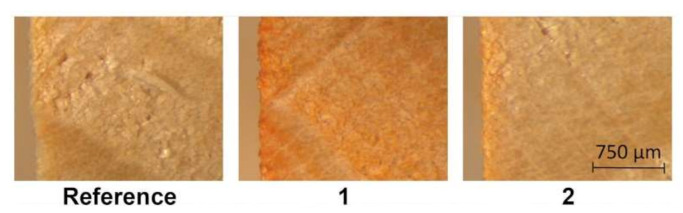
Internal cross-section of a beech reference and two beech samples treated with **DO30Nat** with **Method 1** (application of vacuum-pressure cycle in water coloring solutions) and **Method 2** (single immersion in water coloring solutions), seen using a digital microscope.

**Figure 7:**
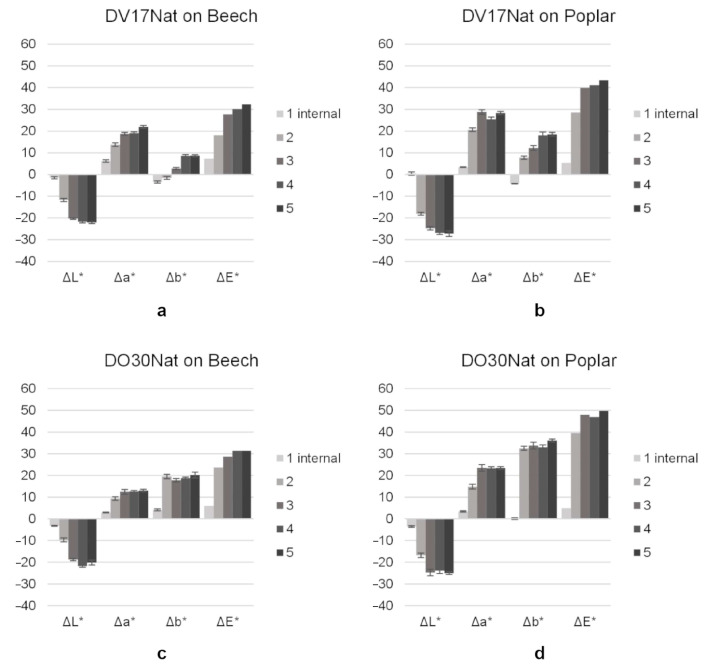
The color difference in terms of ΔL*, Δa*, Δb* and ΔE* between before and after the coloring treatment measured on beech (left) and poplar (right) samples dyed with **DV17Nat** (**a**,**b**) and **DO30Nat** (**c**,**d**) following **Method 1** (application of vacuum-pressure cycle in water coloring solutions, measurements taken from the internal cross-section of the samples), **Method 2** (single immersion in water coloring solutions), **Method 3** (serial immersion in water coloring solutions), **Method 4** (application of Gemma U50 Solas^®^ coloring dispersion by brush) and **Method 5** (application of Resina Plus U49 Solas^®^ on water-based colorations).

**Method 1** gave the best results in terms of surface coloring, but it is also the most demanding in terms of time and the instrumentation used. The good results of this method were probably due to the fact that vacuum-pressure cycles allowed water uptake that did not result in dye penetration because of the high molecule dimension of NDs. This idea is confirmed by the limited penetration of dye inside the specimen shown in Figure 6, and by the colorimetric measurements shown in Figure 7. This type of dye, therefore, does not seem to be suitable for mass coloring and using vacuum techniques for surface coloring is unaffordable.

**Methods 2** and **3**, which are faster and cheaper than **Method 1**, gave less coverage but still a noticeable color (Figure 5). Moving from a single immersion (**Method 2**) to repeated immersions (**Method 3**), an increase in ΔE* values between before and after the coloring treatment registered on the dyed surfaces was observed, particularly in the perceptual lightness difference (ΔL*) (Figure 7). This proved that **Method 3** gave a higher color intensity. This better result is related to the fact that the drying step between the dipping phases allows the formation of a first layer on which the subsequent dye deposits can accumulate [39].

**Method 4** gave similar colorations to those obtained with **Method 3** and, as for the last one, it showed a higher color concentration in earlywood, highlighting the grain of the beech wood. This is due to the different permeability of the wood medium by dispersions, as earlywood has a lower density than latewood. The slightly yellowish tint of Gemma U50 Solas^®^ gave the final colors a warmer hue (Figure 5). This was especially noticeable on the samples treated with **DV17Nat** as the chromophore, whereas those colored with **Method 4** had higher Δb* values and hue angles (h) closer to 90° than those treated with **Method 3**; i.e., they had a final color closer to yellow (Figure 7a,b, Table 1).

The application of a clear top coat on the water-based colorations (**Method 5**) did not change the latter color (Figure 5). Only on the samples treated with **DV17Nat** was there a tendency of the surface coloring towards more yellowish tones (Figure 7a,b, Table 1). This was for the same reason as the stain-based dyes: a yellowish tint of the top coat due to its chemical nature.

### 3.4. Fastness Grade (FG)

The colors obtained with **DO30Nat** and **DV17Nat** as chromophores revealed, in general, high resistance to water washout (Table 2). In fact, when applied in aqueous solutions following **Method 3**, they gave FG values equal to 4 on a scale from 1 to 5 (5 corresponds to a high fixation grade, the absence of water washout).

FG values ranging from 4–5 to 5 were obtained for both beech and poplar samples prepared with **Method 4**. Thus, the resin gave a better fastness grade than the water solutions, as it played a protecting role in the dye and gave hydrophobicity to the treated surfaces, improving the stability of the colors.

The best solution to optimize the color fastness to water washout proved to be the application of two layers of clear top coat on the wooden colored surfaces (**Method 5**) (Table 2). This was due to the presence of the top coat film, which represented a physical barrier that prevented the interaction between water and the underlying color, eliminating any possibility for the latter to be washed away.

### 3.5. Photo-Induced Fading

**Figure 8:**
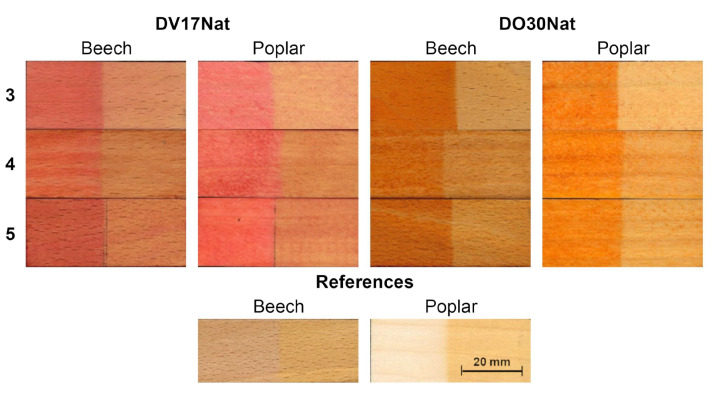
Macroscopical aspect of beech and poplar samples dyed with **DV17Nat** (**top left**) and **DO30Nat** (**top right**) following **Method 3** (serial immersion in water coloring solutions), **Method 4** (application of Gemma U50 Solas^®^ coloring dispersion by brush) and **Method 5** (application of Resina Plus U49 Solas^®^ on water-based colorations), and wooden references (**bottom center**) after 50 h of exposure to artificial aging with a 500 W Xenon lamp. The left part of each sample was covered with aluminum foil, so it was not subjected to photodegradation.

**Figure 9:**
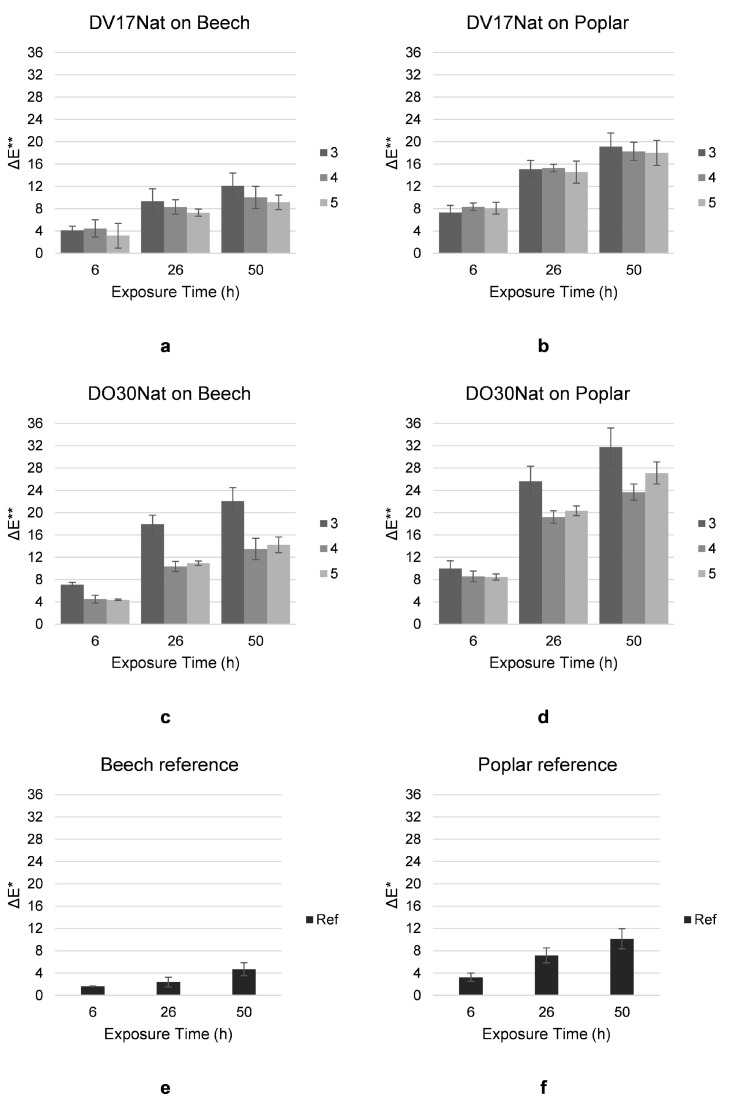
Quantification of color fading after 6, 26 and 50 h of exposure to artificial aging with Xenon lamp on beech (left) and poplar (right) samples treated with **Method 3** (serial immersion in water coloring solutions), **Method 4** (application of Gemma U50 Solas^®^ coloring dispersion by brush) and **Method 5** (application of Resina Plus U49 Solas^®^ on water-based color) with **DV17Nat** (**a**,**b**) and **DO30Nat** (**c**,**d**), and on beech (**e**) and poplar (**f**) references.

The water-based colors (**Method 3**) turned out to be less resistant to UV radiations than the impregnating agent-based ones (**Method 4**), as can be seen from the pictures in Figure 8 and from the quantification of fading after artificial aging in terms of ΔE** values shown in Figure 9.

This is more evident on the poplar samples, because of the greater photosensitivity of this specific wood species. It can be seen, in fact, from the data collected on the reference samples (Figure 9e,f), that, even when untreated, poplar has higher ΔE** values than beech after artificial aging. The addition of two layers of clear top coat (**Method 5**) reduced the color fading on the samples colored with aqueous solutions. In fact, the ΔE** values related to the samples treated with **Method 5** were comparable to those observed on the samples treated with **Method 4**. The better resistance to fading of **Methods 4** and **5** compared to **Method 3** is due to the presence of vegetable oils in both the stain and the clear top coat, which may act, at least in part, as a sacrificial layer and make the associated colorations more photostable [40,41].

The ΔE** values related to color fading recorded here are quite high, even for stain and top-coat-protected surfaces. The color fading of NDs was expected, as their organic nature makes them particularly sensitive to the reaction of photodegradation. However, it appears that the use of vegetable oils, either as a stain or as a top coat, is not sufficient to compensate for this behavior, although it does limit it. Therefore, these dyes seem not to be suitable for outdoor use.

## 4. Conclusions

Natural dyes are an important group of compounds essential for sustainable and cleaner development. Nevertheless, they are characterized by limited color fastness to water washing when applied to wood surfaces unless mordants are used, which can be toxic or harmful, or produce toxic waste. Naturalized dyes (NDs), i.e., synthetic chemical compounds based on the covalent union of a dye species with a natural sugar such as lactose, are a new class of eco-friendly dyes and can be a valid alternative to the extent that they do not require the use of mordants.

In the present work, two water-soluble NDs were studied for dyeing beech and poplar wood. Five application methods were tested, three that exploited the immersion of the wood samples in aqueous solutions and two that involved the application of resin-based products of natural origin by brush. The two NDs used gave stable and optically isotropic solutions and stable coloring dispersions with the resin. Both the water-based solutions and the stains made it possible to obtain vivid and covering surface colors, the application was easy and did not require the addition of chemical auxiliaries. It is believed that dyeing takes place by fixing the chromophore molecules in the porosity of the wood. In fact, the colors obtained with the two NDs were already resistant to water washout when applied in aqueous solutions, while the presence of a resin, either as a stain or as a top coat, gave an even better fastness grade, as it played a protective role with respect to the dyes. However, like natural dyes, NDs are subject to significant color fading when exposed to UV radiation. This behavior was not altered by the use of vegetable oils, although their presence in the stain or as a top coat does limit it. Therefore, these dyes do not seem to be suitable for outdoor use, but rather for indoor use.

## Figures and Tables

**Table 1:** L*C*h values for beech and poplar samples both untreated and dyed with **DV17Nat** and **DO30Nat** following **Method 2** (single immersion in water coloring solutions), **Method 3** (serial immersion in water coloring solutions), **Method 4** (application of Gemma U50 Solas^®^ coloring dispersion by brush) and **Method 5** (application of Resina Plus U49 Solas^®^ on water-based colorations).

Type of Wood	Type of ND	Coloring Method	L*	C*	h (°)
**Beech**	**None**		74.3 ± 0.2	20.1 ± 0.2	57.2 ± 1.1
	**DO30Nat**	**2**	64.6 ± 0.7	41.6 ± 0.3	61.9 ± 1.1
		**3**	55.6 ± 0.3	41.8 ± 0.7	57.1 ± 1.0
		**4**	52.6 ± 0.4	42.7 ± 0.2	57.6 ± 0.2
		**5**	54.2 ± 0.9	44.1 ± 1.0	58.1 ± 0.5
	**DV17Nat**	**2**	62.6 ± 0.5	28.7 ± 0.3	33.2 ± 1.1
		**3**	54.0 ± 0.1	35.3 ± 0.4	34.6 ± 0.3
		**4**	52.7 ± 0.4	39.1 ± 0.3	41.4 ± 0.6
		**5**	52.2 ± 0.2	41.3 ± 0.3	38.8 ± 0.5
**Poplar**	**None**		82.3 ± 0.4	8.7 ± 0.5	57.2 ± 1.1
	**DO30Nat**	**2**	65.5 ± 0.6	44.4 ± 0.6	63.9 ± 0.8
		**3**	57.6 ± 1.1	49.8 ± 0.4	55.5 ± 1.8
		**4**	58.4 ± 0.9	49.0 ± 0.5	55.3 ± 0.8
		**5**	57.3 ± 0.2	51.7 ± 0.4	58.1 ± 0.5
	**DV17Nat**	**2**	64.2 ± 0.2	29.5 ± 0.2	30.7 ± 1.1
		**3**	57.6 ± 0.4	38.8 ± 0.3	30.2 ± 1.4
		**4**	55.5 ± 0.3	39.4 ± 0.4	40.0 ± 1.7
		**5**	55.1 ± 0.9	41.9 ± 0.5	38.0 ± 0.5

**Table 2:** Quantification of color migration on glass fiber sheets (ΔE*) during the color fastness tests [36] and relative fastness grade (FG) of beech and poplar samples treated with **Method 3** (serial immersion in water coloring solutions), **Method 4** (application of Gemma U50 Solas^®^ coloring dispersion by brush) and **Method 5** (application of Resina Plus U49 Solas^®^ on water-based color) with **DV17Nat** and **DO30Nat**.

Coloring Method	DO30Nat				DV17Nat			
	Beech		Poplar		Beech		Poplar	
	ΔE*	FG	ΔE*	FG	ΔE*	FG	ΔE*	FG
**3**	3.2 ± 0.8	4	2.7 ± 0.7	4	3.4 ± 0.8	4	3.0 ± 0.9	4
**4**	0.5 ± 0.2	5	0.5 ± 0.1	5	1.7 ± 0.4	4–5	1.7 ± 0.4	4–5
**5**	0.2 ± 0.1	5	0.5 ± 0.3	5	0.6 ± 0.1	5	0.6 ± 0.2	5

## Data Availability

Not applicable.

## Supplementary Materials

The following supporting information can be downloaded at: https://www.mdpi.com/article/10.3390/polym15173632/s1: experimental procedure for the synthesis of DO30Nat and DV17Nat, Scheme S1: Synthesis of the protected (piperazin-6′-yl)lactose 4; Scheme S2: Synthesis of the ND DO30Nat; Scheme S3: Synthesis of the ND DV17Nat. References [42,43,44,45,46,47] are cited in the supplementary materials.
