# Supplementary material for: Naturalized Dyes: A New Opportunity for the Wood Coloring
Source: Polymers (Basel). 2023 Sep 1;15(17):3632. doi: 10.3390/polym15173632 (PMC10490190; doi:10.3390/polym15173632)
Supplement: Supplementary file 1 [file polymers-15-03632-s001.zip › polymers-2528949-supplementary.pdf]

SUPPLEMENTARY MATERIAL

Naturalized dyes: a new opportunity for the wood coloring

Laura Vespignani [1,3]*, Marco Bonanni [1], Marco Marradi [1], Benedetto Pizzo [2], Roberto Bianchini [1], Giacomo Goli [3]

[1] Department of Chemistry "Ugo Schiff", University of Florence, Via della Lastruccia 3-13, 50019, Sesto Fiorentino (FI), Italy; [2] CNR-Institute of Bioeconomy (IBE CNR), Via Madonna del Piano, 10. 50019, Sesto Fiorentino (FI), Italy; [3] Department of Agriculture, Food, Environment and Forestry (DAGRI), University of Florence, Piazzale delle Cascine 18, 50144, Florence, Italy

*Corresponding author: Laura Vespignani, laura.vespignani@unifi.it

1. Materials used for the chemical synthesis of the naturalized dyes (NDs) Disperse Orange 30 (DO30Nat) and Disperse Violet 17 (DV17Nat)

Commercially available reagents and solvents were purchased from Sigma-Aldrich and were used without further purification. The notation PE refers to the petroleum ether fraction boiling between 40 and 60 °C. Thin-layer chromatography (TLC) analysis for the determination of the retention factor (R_f) was performed using aluminium foils coated with silica gel 60 matrix with fluorescent indicator F_{254}. TLC development was performed under UV (254 and 366 nm) or visible light, followed by treatment with an acid solution of *p*-anisaldehyde or a basic solution of $KMnO_4$ and heating. Melting points (mp) were recorded on a SMP3 apparatus from Stuart Scientific. Optical rotations were measured on a Jasco DIP-370 polarimeter using a 100 mm path-length cell at 589 nm. ^{1}H-NMR spectra were recorded at 400 MHz on an NMR Varian Mercury Plus 400 instrument. The NMR samples were prepared by dissolving the analyte (8 mg) in 0.8 mL of the selected deuterated solvent. WILMAD WG-1228 Class B Borosilicate Glass 5 mm Economy NMR Tube, 7" (400 MHz) were used. Chemical shifts (δ) are reported in parts per million (ppm) and referenced to the solvent residual peaks: 7.27 ppm for $CDCl_3$, 2.50 ppm for DMSO-d_6 and 4.79 ppm for D_2O. Multiplicity is indicated by s = singlet, d = doublet, dd = doublet of doublets, t = triplet, dt = doublet of triplets, td = triplet of doublets, q = quartet, br = broad signal, m = multiplet. Coupling constants *J* are reported in Hertz. Mass spectra were recorded on a Thermo Scientific LCQ-Fleet mass spectrometer under electrospray ionisation (ESI, +c technique). Preparation of the samples involved the dissolution of the analyte (approximately 1 mg) in 1 mL of methanol, and further 1 to 10 dilution before direct infusion. Mass spectrometric analysis data are quoted in the *m*/*z* form. FT IR spectra were recorded with a Shimadzu FT-IR spectrophotometer, IR Affinity-1S model. Spectra of solid samples were recorded in ATR mode. Peaks appearance is indicated by w = weak, m = medium, s = strong, brd = broad.

2. Synthesis of lactose derivative 4 (Scheme S1)

The reaction of commercially available lactose **1** with 2,2-dimethoxypropane (DMP) afforded the protected diol **2**, whose 6′ and 2′ positions are available for further elaboration, following a reported procedure. [42] Regioselective tosylation of the primary alcohol (position 6′) afforded compound **3**, [43] which was then subjected to a bimolecular nucleophilic substitution using an excess of piperazine (replacement of the tosyl group by the piperazinyl moiety) according to a patented procedure. [26] Compound **4** was obtained in 62% overall yield for three steps (Scheme S1). The piperazine derivative **4** was then used to functionalize the commercially available dyes Disperse Orange 30 (section 3) and Disperse Violet 17 (section 4).

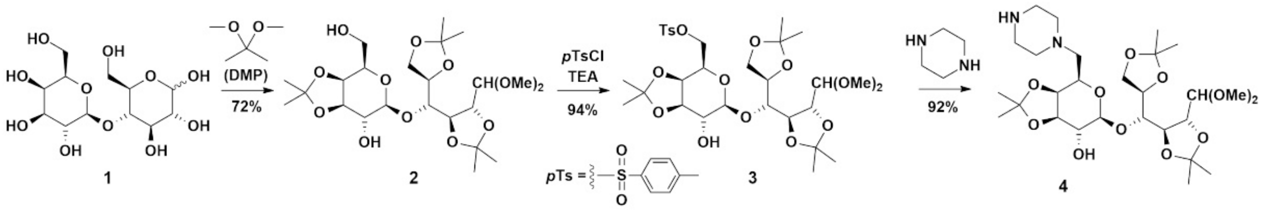

Scheme S1. Synthesis of the protected (piperazin-6′-yl)lactose **4**.

2.1. *Experimental procedure for the synthesis of lactose derivative* **2**

Lactose monohydrate (170.0 g, 0.472 mol) was suspended in dimethoxypropane (1.27 kg), and *p*-toluensulphonic acid monohydrate (7.2 g, 0.038 mol) was added. The mixture was heated at 65 °C for 6 h. TLC analysis (EtOAc : PE = 70 : 30) revealed the disappearance of lactose and a new major spot (R_f = 0.60). The clear solution was cooled to 20 °C; triethylamine (6.2 g, 0.061 mol) was added, and the solvent was evaporated under reduced pressure. The crude material was dissolved in dichloromethane (0.80 kg), and the resulting solution was treated with aq. HCl (0.5 M, 181.4 g solution) for 2 min under mechanical stirring. Next, a solution of NH_4OH (1.0 M, 88.2 g solution) was added, and the organic phase was separated, washed with water (0.30 kg) and concentrated to 1/4 of its volume. The syrup-like material was added with ethyl acetate (80.0 g). Next, PE (0.65 kg) was added in portions to obtain a slurry, which was kept under stirring overnight. The suspended solid was filtered, washed with PE (2 × 0.20 kg) and dried at 40 °C to recover compound **2** (170.0 g, ~70%) as an off-white powder, with mp 95–96 °C (lit. [44] 95–98 °C); ^{1}H-NMR δ_H (400 MHz, $CDCl_3$): 4.58 (1H, dd, *J* 8.0 and *J′* 6.8 Hz), 4.41 (1H, d, *J* 8.4 Hz), 4.35 (1H, d, *J* 6.4 Hz), 4.34–4.30 (1H, m), 4.19 (1H, dd, *J* 9.2 and *J′* 5.2 Hz), 4.08–3.98 (4H, m), 3.95–3.88 (2H, m), 3.80 (1H, dt, *J* 9.2 and *J′* 2.4 Hz), 3.64 (1H, td, *J* 12.0 and 2.4 Hz), 3.54–3.50 (1H, m) 3.48 (3H, s), 3.47 (3H, s), 3.41 (1H, dd, *J* 12.0 and *J′* 1.6 Hz), 2.91 (1H, d, *J* 1.6 Hz), 1.48 (6H, s), 1.37 (6H, s), 1.31 (3H, s), 1.30 (3H, s); ESI (*m*/*z*, +c): 531.4 $[M+Na]^+$, 1038.5 $[2M+Na]^+$.

2.2. *Experimental procedure for synthesis of lactose derivative* **3**

Compound **2** (170.0 g, 0.330 mol) was dissolved in dichloromethane (0.72 kg), and the solution was cooled to 0 °C. Triethylamine (54.4 g, 0.530 mol) was added, followed by *N*,*N*-dimethylaminopyridine (4.1 g, 0.030 mol). The mixture was stirred for 10 min, and *p*-toluensulfonylchloride (73.0 g, 0.380 mol) was added portionwise. The reaction was warmed to 20 °C within an hour and kept at this temperature for 2 h. Then, triethylamine (10.0 g, 0.099 mol) was added, and stirring continued for a further 3 h, after which TLC analysis (EtOAc : PE = 60 : 40) confirmed the absence of compound **2** and the appearance of a new spot (R_f = 0.76). Thus, aq. Na_2CO_3 (39.0 g/0.30 kg water) was added, and the two-phase mixture was stirred at 500 rpm overnight using a mechanical stirrer. Then, the organic phase was separated, washed sequentially with an acid solution (25.8 g citric acid monohydrate/0.26 kg water) and water (0.20 kg), and evaporated to dryness. The crude material (207.0 g) was dissolved in acetonitrile (0.32 kg) containing triethylamine (0.69 g, 0.007 mol) and stored at 4 °C for the next step. Compound **3** was obtained as an off-white foamy solid, with mp 54–55 °C (lit. [45] 57–59 °C); ^{1}H-NMR δ_H (400 MHz, $CDCl_3$): 7.81 [2H, d (AA′XX′), *J* 8.2 Hz], 7.36 [2H, d (AA′XX′), *J* 8.2 Hz], 4.46–4.40 (3H, m), 4.30 (1H, td, *J* 6.8 and *J′* 2.4 Hz), 4.26 (1H, dd, *J* 16.0 and *J′* 5.6 Hz), 4.20–4.12 (2H, m), 4.10–4.05 (3H, m), 4.03–3.99 (2H, m), 3.94 (1H, dd, *J* 7.0 and *J′* 1.4 Hz), 3.50 (1H, t (br), *J* 6.8 Hz), 3.45 (3H, s), 3.43 (3H, s), 3.38 (1H, d, *J* 1.2 Hz), 2.47 (3H, s), 1.45 (3H, s), 1.39 (3H, s), 1.37 (3H, s), 1.34 (3H, s), 1.28 (3H, s); ESI (*m*/*z*, +c): 685.5 $[M+Na]^+$.

2.3. *Experimental procedure for synthesis of lactose derivative* **4**

Piperazine (240.0 g, 2.786 mol) was suspended in acetonitrile (1.60 kg) and heated at 60 °C. A solution of compound **3** (207.0 g, 0.312 mol) in acetonitrile (0.32 kg) was added dropwise within 1.5 h whilst bringing the internal temperature up to the boiling point of the solvent (83–84 °C). The mixture was stirred for a further 3.5 h, when TLC analysis (EtOAc : PE = 60 : 40) indicated the disappearance of tosylate **3**. The reaction was cooled to 20 °C and the solvent evaporated. The crude material was dissolved in dichloromethane (2.50 kg), and the resulting solution was washed with water (0.95 kg). After separation of the organic phase, evaporation of the solvent furnished compound **4** (165.0 g, 92%) as an off-white foamy solid with mp 60–62 °C (lit. [26] 62–64 °C); ^{1}H-NMR δ_H (400 MHz, $CDCl_3$): 4.41–4.38 (1H, m), 4.38 (1H, d, *J* 8.0 Hz), 4.33 (1H, d, *J* 6.4 Hz), 4.27 (1H, td, *J* 2.4

and J' 6.8 Hz), 4.17–4.08 (3H, m), 4.04–4.00 (2H, m), 3.90 (1H, dd, J 1.2 and J' 7.2 Hz), 3.85–3.82 (1H, m), 3.56–3.52 (1H, m), 3.46 (3H, s), 3.45 (3H, s), 3.44 (1H, d, J 2.8 Hz), 2.95–2.92 (4H, m), 2.79–2.68 (2H, m), 2.65–2.43 (5H, m), 1.51 (3H, s), 1.50 (3H, s), 1.39 (3H, s), 1.38 (3H, s), 1.35 (3H, s), 1.34 (3H, s); ESI (*m*/*z*, +c): 577.5 $[M+H]^+$.

3. Synthesis of Naturalized Disperse Orange 30 (DO30Nat) (Scheme S2)

The synthesis of **DO30Nat** was carried out as reported in the literature. [27] Briefly: Dye **5**, which was prepared from chromophore DO30, [26] was deprotected in basic conditions and the crude compound **6** was reacted with succinic anhydride. The free carboxylic acid moiety of the resulting compound **7** was activated with 4-(4,6-dimethoxy-1,3,5-triazin-2-yl)-4-methylmorpholinium chloride (DMTMM) in mild conditions. [46] Final deprotection of the acetonide moieties was carried out in acid conditions, to restore the original structure of the lactose moiety within **DO30Nat** (Scheme S2). [26]

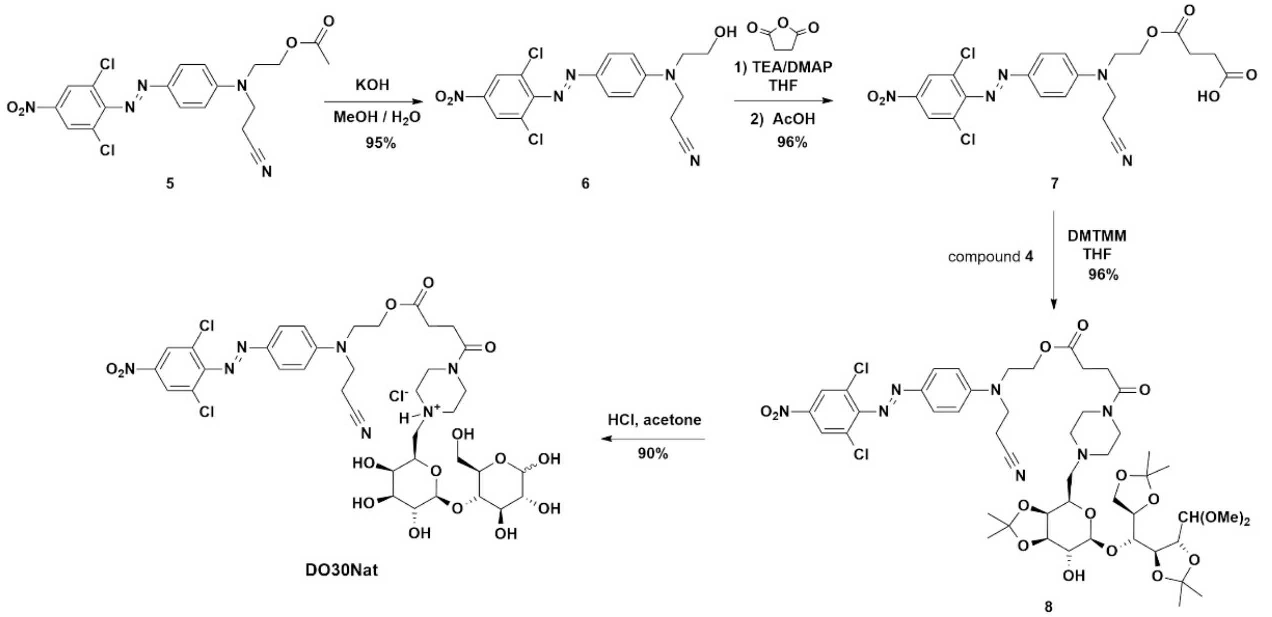

Scheme S2. Synthesis of the ND **DO30Nat**.

3.1. *Experimental procedure for the synthesis of compound* 6

Commercially available Disperse Orange 30 (**5**) (10.0 g, 22.2 mmol) was dissolved in methanol (80 mL) and an aq. solution (40 mL) of potassium carbonate (3.7 g, 26.8 mmol) was added portionwise at 0 °C. The resulting mixture was stirred for 20 min, it was warmed to 20 °C and stirred overnight. The solvent was evaporated under reduced pressure and the crude residue was triturated in methanol and water (150 mL, 1:5 v/v.). The fine suspension was filtered under suction and the press cake was washed with water and dried in air, to obtain compound **6** (8.6 g, 95%) as dark orange solid, mp 125–127 °C; ^{1}H-NMR δ_H (400 MHz, $CDCl_3$): 8.28 (2H, s), 7.96 (2H, d, J 9.3 Hz), 6.83 (2H, d, J 9.3 Hz), 4.01–3.88 (4H, m), 3.75 (2H, t, J 5.4 Hz), 2.79 (2H, t, J 6.9 Hz); ESI (m/z, +c): 408.2 (100%) $[M+H]^+$ 410.1 (51%) $[M+H]^+$.

3.2. *Experimental procedure for the synthesis of compound* 7

An ice-cooled solution of compound **6** (14.3 g, 35.0 mmol), dihydrofuran-2,5-dione (4.2 g, 42.0 mmol) and *N*,*N*-dimethylpyridin-4-amine (0.4 g, 3.3 mmol) in tetrahydrofuran (170 mL) was treated with triethylamine (4.6 g, 45.5 mmol) at 0 °C dropwise. The resulting mixture was warmed to 20 °C overnight: then, acetic acid (10.5 g, 174.9 mmol) was added and the whole was stirred for 1 h. The solution was washed with water and brine: the organic phase was then separated, dried over anhydrous sodium sulphate and filtered. The filtrate was concentrated in vacuo to yield compound **7** (17.9 g, 96%) as dark red solid, mp 125–127 °C; ^{1}H-NMR δ_H (400 MHz, $CDCl_3$): 8.26 (2H, s), 7.95 (2H, d, J 9.3 Hz), 6.82 (2H, d, J 9.3 Hz), 4.38 (2H, t, J 5.7 Hz), 3.88–3.80 (4H, m), 2.72 (2H, t, J 6.9 Hz), 2.71–2.59 (4H, m); ESI (m/z, -c): 506.0 (14%) $[M-H]^-$, 508.0 (7%) $[M-H]^-$, 1013.0 (77%) $[2M-H]^-$, 1014.9 (100%) $[2M-H]^-$, 1017.1 (58%) $[2M-H]^-$, 1019.1 (9%) $[2M-H]^-$.

*3.3. Experimental procedure for the synthesis of compound **8***

A solution of compound **7** (4.1 g, 8.0 mmol) and compound **4** (5.3 g, 9.2 mmol) in anhydrous tetrahydrofuran (100 mL) was stirred for 4 h at 20 °C under a nitrogen atmosphere. DMTMM (2.4 g, 8.7 mmol) was added portionwise and the resulting mixture was stirred overnight. The solvent was evaporated in vacuo and the residue was partitioned between ethyl acetate and water. The organic phase was separated, washed with brine, dried over anhydrous sodium sulphate and filtered. The filtrate was concentrated under reduced pressure, to yield compound **8** (8.2 g, 96%) as foamy orange solid, mp 133–134.5 °C; ^{1}H-NMR δ_H (400 MHz, $CDCl_3$): 8.28 (2H, s), 7.96 (2H, d, *J* 9.3 Hz), 6.83 (2H, d, *J* 9.3 Hz), 4.40–4.25 (6H, m), 4.18–4.00 (5H, m), 3.96–3.82 (7H, m), 3.62–3.52 (3H, m), 3.48–3.41 (2H, m), 3.46 (3H, s), 3.45 (3H, s), 2.78–2.73 (4H, m), 2.64–2.43 (8H, m), 1.51 (3H, s), 1.50 (3H, s), 1.40 (3H, s), 1.38 (3H, s), 1.35 (3H, s), 1.33 (3H, s); ESI (m/z, +c): 1066.8 (100%) $[M+H]^+$,1088.6 (10%) $[M+Na]^+$.

*3.4. Experimental procedure for the synthesis of compound **DO30Nat***

A solution of compound **8** (8.5 g, 8.0 mmol) in acetone (100 mL) was cooled to 0 °C and an ice-cooled solution of aq. hydrochloric acid (1.7 mL, 37% w/w, 20.0 mmol) in acetone (15 mL) was added dropwise. The resulting mixture was warmed to 20 °C overnight, after which the solvent was decanted. The precipitated residue was triturated in fresh acetone three times and then it was dried in vacuo. Compound ***DO30Nat*** (6.8 g, 90%) was obtained as dark orange solid, mp 174–183 °C (dec.); λ_{max} (ε) (H_2O): 441 nm (16300 $M^{-1}cm^{-1}$); ^{1}H-NMR δ_H (400 MHz, DMSO-d_6): 8.44 (2H, s), 7.85 (2H, d, *J* 9.3 Hz), 7.06 (2H, d, *J* 9.3 Hz), 5.13–4.52 (8H, m), 4.43–4.21 (4H, m), 4.14–3.95 (2H, m), 3.93–3.40 (13H, m), 3.37–2.92 (11H, m), 2.90–2.82 (2H, s), 2.70–2.60 (1H, m), 2.58–2.53 (1H, s); ESI (m/z, +c): 900.2 (100%) $[M+H]^+$, 902.3 (89%) $[M+H]^+$, 904.0 (43%) $[M+H]^+$, 922.3 (18%) $[M+Na]^+$, 924.2 (12%) $[M+Na]^+$.

4. Synthesis of Naturalized Disperse Violet 17 (DV17Nat) (Scheme S3)

DV17Nat was prepared following a synthetic strategy similar to that used to synthesize compound **DO30Nat** (Scheme S3). [26] Compound **9** was reacted with ethyl bromoacetate. [47] The resulting ester derivative **10** was hydrolyzed to acid **11**, which was further converted to **12** following the route described above to obtain compound **8**. Final deprotection of the acetonide moieties was carried out in acid conditions, to restore the original structure of the lactose moiety within **DV17Nat.** [26]

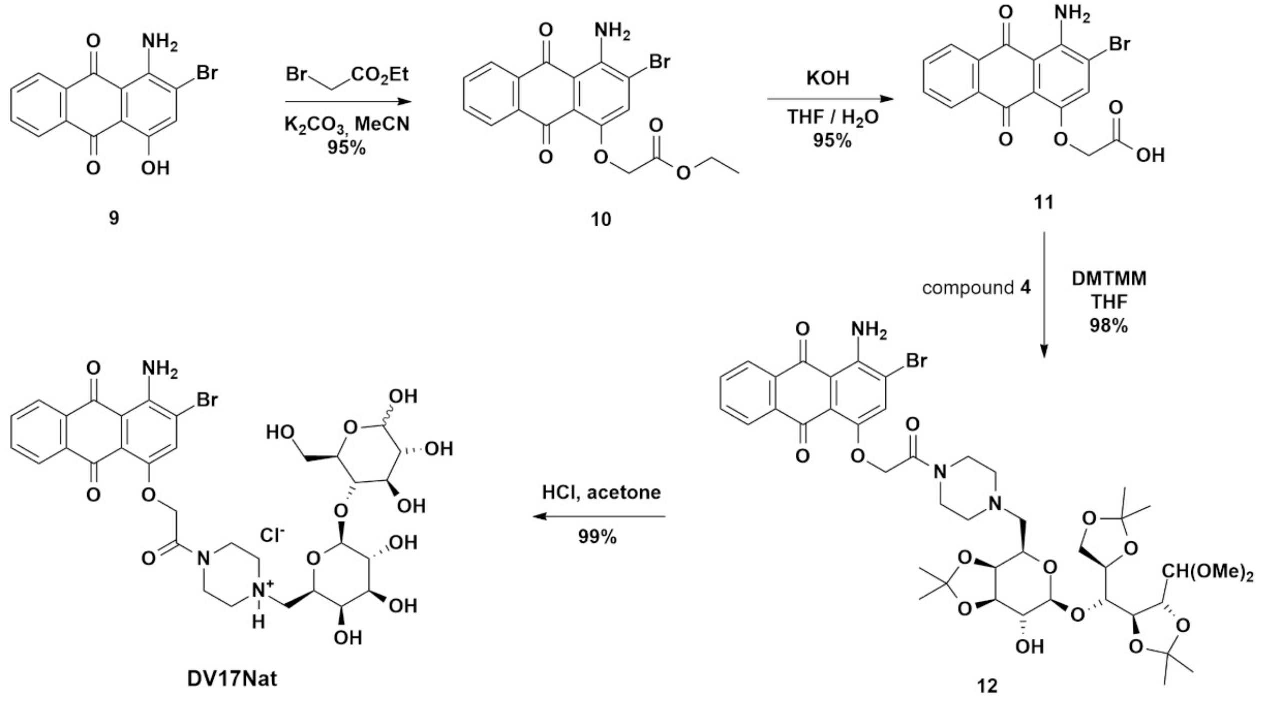

Scheme S3. Synthesis of the ND **DV17Nat**.

4.1. *Experimental procedure for the synthesis of compound* **10**

Commercially available Disperse Violet 17 (**9**) (6.4 g, 20.1 mmoI) was dissolved in acetonitrile (100 mL), potassium carbonate (6.1 g, 44.1 mmol) and ethyl bromoacetate (4.0 mL, 36.0 mmol) were added. The resulting mixture was refluxed for 3 h. The slurry was cooled to 20 °C, poured in a 0.1 M HCl solution (700 mL) and filtered under suction. The press cake was washed with water and dried in air, to obtain compound **10** (7.7 g, 95%) as red solid, mp 134–135 °C; ^{1}H-NMR δ_H (400 MHz, $CDCl_3$): 8.25–8.18 (2H, m), 7.75–7.71 (3H, m), 4.71 (2H, s), 4.29 (2H, q, *J* 7.2 Hz), 1.32 (3H, t, *J* 7.2 Hz); IR (neat): 3399 (m), 3283 (m), 1740 (s), 1662 (m), 1589 (s), 1522 (s), 1263 (m), 1192 (s), 1022 (m), 737 (m) cm^{-1}; ESI (m/z, +c): 404.0 (100%) $[M+H]^+$ 406.0 (97%) $[M+H]^+$.

4.2. *Experimental procedure for the synthesis of compound* **11**

Compound **10** (7.7 g, 19.1 mmoI) was dissolved in tetrahydrofuran (60 mL) and treated with 1.0 M aq. potassium hydroxide (38 mL) at 0 °C. The resulting mixture was warmed to 20 °C overnight. Aq. 1.0 M hydrochloric acid was added to bring the pH to 2-3 and the fine slurry was filtered under suction. The press cake was washed with water and dried in air. Compound **11** (8.6 g, 95%) was obtained as red solid, mp 114–123 °C (dec.); ^{1}H-NMR δ_H (400 MHz, DMSO-d_6): 8.18–8.00 (2H, m), 7.76–7.73 (3H, m), 4.67 (2H, s), 3.75 (2H, bs); IR (neat): 3401 (w), 3280 (w), 3210 (m, brd), 1761 (s), 1638 (m), 1587 (s), 1522 (s), 1396 (m), 1267 (s), 1213 (s), 1018 (s), 737 (m) cm^{-1}; ESI (m/z, -c): 770.7 (51%) $[2M+Na]^-$, 772.7 (100%) $[2M+Na]^-$, 774.7 (40%) $[2M+Na]^-$.

4.3. *Experimental procedure for the synthesis of compound* **12**

A solution of compound **11** (9.4 g, 25.0 mmoI) and compound **4** (16.6 g, 28.8 mmoI) in anhydrous tetrahydrofuran (300 mL) was stirred for 4 h at 20 °C under a nitrogen atmosphere. DMTMM (7.9 g, 28.8 mmoI) was added portionwise and the resulting mixture was stirred overnight. The solvent was evaporated in vacuo and the residue was partitioned between ethyl acetate and water. The organic phase was separated, washed with brine, dried over anhydrous sodium sulphate and filtered. The filtrate was concentrated under reduced pressure, to yield compound **12** (22.9 g, 98%) as foamy red solid, mp 108–110 °C; ^{1}H-NMR δ_H (400 MHz, $CDCl_3$): 8.27–8.15 (2H, m), 7.79–7.71 (3H, m), 4.81 (2H, s), 4.39–4.32 (2H, m), 2.31–4.23 (2H, m), 4.17–4.00 (5H, m), 3.97–3.82 (4H, m), 3.76–3.56 (4H, m), 3.55–3.49 (2H, m), 3.45 (3H, s), 3.44 (3H, s), 2.81–2.67 (2H, m), 2.66–2.44 (4H, m), 1.50 (3H, s), 1.49 (3H, s), 1.38 (3H, s), 1.36 (3H, s), 1.33 (3H, s), 1.31 (3H, s); IR (neat): 3390 (w), 3280 (w), 3934 (w), 1651 (s), 1524 (m), 1368 (m), 1211 (s), 1061 (s), 1018 (s), 854 (m), 738 (w) cm^{-1}; ESI (m/z, +c): 934.2 (100%) $[M+H]^+$,936.3 (37%) $[M+H]^+$, 956.3 (55%) $[M+Na]^+$,958.3 (60%) $[M+Na]^+$.

4.4. *Experimental procedure for the synthesis of compound* ***DV17Nat***

A solution of compound **12** (22.9 g, 24.5 mmoI) in acetone (240 mL) was cooled to 0 °C and an ice-cooled solution of aq. hydrochloric acid (9.1 mL, 37% w/w, 73.4 mmoI) in acetone (30 mL) was added dropwise. The resulting mixture was warmed to 20 °C overnight, after which the solvent was decanted. The precipitated residue was triturated with fresh acetone three times and then it was dried in vacuo. Compound ***DV17Nat*** (15.5 g, 99%) was obtained as dark solid, mp 198–213 °C (dec.); λ_{max} (ε) (H_2O): 501 nm (3020 $M^{-1}cm^{-1}$); ^{1}H-NMR δ_H (400 MHz, DMSO-d_6): 8.18–8.04 (2H, m), 7.88–7.80 (3H, m), 5.05–5.97 (2H, m), 4.92–4.88 (1H, m), 4.78–4.76 (1H, m), 4.41–4.21 (6H, m), 4.04–3.94 (2H, m), 3.87–2.92 (24H, m), 2.48 (2H, s), 2.07 (1H, s); IR (neat): 3292 (m, brd), 1651 (m), 1589 (m), 1526 (m), 1396 (w), 1267 (m), 1217 (m), 1016 (s), 727 (w) cm^{-1}; ESI (m/z, +c): 768.1 (42%) $[M+H]^+$, 770.1 (44%) $[M+H]^+$, 790.1 (100%) $[M+Na]^+$, 792.1 (84%) $[M+Na]^+$.

5. References

26. Bianchini, R.; Corsi, M.; Bonanni, M. WIPO Patent WO2014177528A1 **2014**

27. Pellegrini, D.; Corsi, M.; Bonanni, M.; Bianchini, R.; D'Ulivo, A.; Bramanti, E. Study of the interaction between collagen and naturalized and commercial dyes by Fourier transform infrared spectroscopy and thermogravimetric analysis. *Dyes Pigm* **2015**, *116*, 65-73

42. Barili, P.L.; Catelani, G.; D'Andrea, F.; De Rensis, F.; Falcini, P. Improved preparation of 2,3:5,6:30,40-tri-O-isopropylidenelactose dimethyl acetal and its 60-O-(1-methoxy-1-methylethyl) derivative. *Carbohydr Res* **1997**, *298*, 75-84

43. Kabalka, G.W.; Varma, M.; Varma, R.S.; Srivastava, P.C.; Knapp Jr, F.F. The tosylation of alcohols. *J Org Chem* **1986**, *51*, 2386-2388

44. Attolino, E.; Catelani, G.; D'Andrea, F. Regiospecific synthesis of 4-deoxy-D-*threo*-hex-3-enopyranosides by simultaneous activation–elimination of the talopyranoside axial 4-OH with the NaH/$Im_{S2}O_2$ system: manifestation of the stereoelectronic effect. *Eur J Org Chem* **2006**, *23*, 5279-5292
45. Catelani, G.; Corsaro, A.; D'Andrea, F.; Mariani, M.; Pistarà, V.; Vittorino, E. Convenient preparation of l-arabinohexos-5-ulose derivatives from lactose. *Carbohydr Res* **2003**, *338*, 2349-2358
46. Kunishima, M.; Kawachi, C.; Iwasaki, F.; Terao, K.; Tani, S. Synthesis and characterization of 4-(4,6-dimethoxy-1,3,5-triazin-2-yl)-4-methylmorpholinium chloride (DMT-MM). *Tetrahedron Lett* **1999**, *40*, 5327-5330
47. Baughman, T.W.; Sworen, J.C.; Wagener, K.B. The facile preparation of alkenyl metathesis synthons. *Tetrahedron* **2004**, *60*, 10943-10948
